# Ca^2+^ current facilitation is CaMKII-dependent and has arrhythmogenic consequences

**DOI:** 10.3389/fphar.2014.00144

**Published:** 2014-06-17

**Authors:** Donald M. Bers, Stefano Morotti

**Affiliations:** Department of Pharmacology, University of California DavisDavis, CA, USA

**Keywords:** CaMKII, calcium channel, calcium current inactivation, calcium current facilitation, calcium current staircase

## Abstract

The cardiac voltage gated Ca^2+^ current (I_Ca_) is critical to the electrophysiological properties, excitation-contraction coupling, mitochondrial energetics, and transcriptional regulation in heart. Thus, it is not surprising that cardiac I_Ca_ is regulated by numerous pathways. This review will focus on changes in I_Ca_ that occur during the cardiac action potential (AP), with particular attention to Ca^2+^-dependent inactivation (CDI), Ca^2+^-dependent facilitation (CDF) and how calmodulin (CaM) and Ca^2+^-CaM dependent protein kinase (CaMKII) participate in the regulation of Ca^2+^ current during the cardiac AP. CDI depends on CaM pre-bound to the C-terminal of the L-type Ca^2+^ channel, such that Ca^2+^ influx and Ca^2+^ released from the sarcoplasmic reticulum bind to that CaM and cause CDI. In cardiac myocytes CDI normally pre-dominates over voltage-dependent inactivation. The decrease in I_Ca_ via CDI provides direct negative feedback on the overall Ca^2+^ influx during a single beat, when myocyte Ca^2+^ loading is high. CDF builds up over several beats, depends on CaMKII-dependent Ca^2+^ channel phosphorylation, and results in a staircase of increasing I_Ca_ peak, with progressively slower inactivation. CDF and CDI co-exist and in combination may fine-tune the I_Ca_ waveform during the cardiac AP. CDF may partially compensate for the tendency for Ca^2+^ channel availability to decrease at higher heart rates because of accumulating inactivation. CDF may also allow some reactivation of I_Ca_ during long duration cardiac APs, and contribute to early afterdepolarizations, a form of triggered arrhythmias.

## Introduction

The cardiac L-type Ca^2+^ channel (LTCC) current (I_Ca_) is an important contributor to overall cardiac electrophysiology and arrhythmias, excitation-contraction coupling (ECC; it causes further intracellular Ca^2+^ release and activation of the myofilaments), mitochondrial energy regulation, cell death and transcriptional regulation (Bers, 2008). I_Ca_ is mainly via the Cav1.2 α1 LTCC isoform, although the Cav1.3 isoform is expressed in some atrial cells (especially pacemaker cells). That pore-forming α1 subunit also carries the intrinsic voltage-dependent gating properties (Perez-Reyes et al., 1989) and many key regulatory sites. However, the mature LTCC in heart is a complex containing also a β as well as an α2-δ subunit that influence LTCC trafficking and gating (Shirokov et al., 1998; Bichet et al., 2000; Wei et al., 2000; Dzhura and Neely, 2003). Cav1.2 has four major domains (I-IV), each of which contains six transmembrane segments (S1-S6), where positive charges in the S4 segments participate as voltage sensors and the S5-S6 loop is the locus of the ion-conducting pore (Bers, 2001).

The rapid upstroke or phase 0 of the cardiac action potential (AP) is driven by Na^+^ current (I_Na_) in most cardiac myocytes, and causes voltage-dependent activation of I_Ca_. In pacemaker cells in the sino-atrial and atrio-ventricular node, it is I_Ca_ activation that is responsible for the rapid upstroke of the AP. I_Ca_ activation is a bit slower than I_Na_ activation, but starts early during the cardiac AP. The early repolarization phase of the AP (phase 1) can enhance I_Ca_ because of an increase in electrochemical driving force, i.e., membrane potential (E_m_) is further from the Ca^2+^ equilibrium potential (E_Ca_; Sah et al., 2002). However, both depolarization and the rise in local intracellular [Ca^2+^] ([Ca^2+^]_i_) begin the processes of voltage- and Ca^2+^-dependent inactivation (VDI and CDI), which continues during the plateau phase of the AP (phase 2) causing a progressive decrease in I_Ca_. As rapid and terminal AP repolarization ensue (phase 3) the LTCC undergoes de-activation, but then recovery from inactivation is both time and E_m_-dependent. Thus, for LTCC to recover full availability between beats, some time must elapse and that recovery time depends on E_m_ (e.g., at −80 and −50 mV the time constant is about 100 and 400 ms, respectively).

I_Ca_ amplitude and gating properties are influenced by myriad regulatory pathways, but here we will focus on the Ca^2+^-dependent mechanisms that shape the I_Ca_ occurring during the AP in ventricular myocytes. Hence, this review will describe how the Ca^2+^ sensing protein calmodulin (CaM) mediates CDI, and is involved in the activation of CaMKII, a serine/threonine-specific protein kinase which is a key mediator of ECC. Note that, although CaMKII activation can also be Ca^2+^-independent (see accompanying article by Erickson, 2014), here we will focus on the main activation mechanism, which is Ca^2+^/CaM dependent. Moreover, the particular structure of this kinase (well described in this series by Pellicena and Schulman, 2014) confers to CaMKII the ability to integrate oscillatory Ca^2+^ signals, because CaMKII activity depends on both frequency and duration of previous Ca^2+^/CaM pulses (De Koninck and Schulman, 1998; Saucerman and Bers, 2008). We will show how the CaMKII-dependent LTCC phosphorylation mediates the Ca^2+^-dependent facilitation (CDF) of I_Ca_, and how this process can eventually lead to E_m_ or Ca^2+^ instabilities in ventricular myocytes.

## Ca^2+^- vs. E_m_-dependent inactivation of I_Ca_

Inactivation of I_Ca_ is driven by VDI and CDI (Kass and Sanguinetti, 1984; Lee et al., 1985; Hadley and Hume, 1987). Several studies have shown that the Ca^2+^-sensing protein CaM mediates CDI by interacting with the carboxyl tail of the LTCC α1 subunit (Zuhlke and Reuter, 1998; Peterson et al., 1999; Qin et al., 1999; Zuhlke et al., 1999; Pate et al., 2000), a cytoplasmic region that contains an EF-hand region and an IQ motif. At rest, CaM is pre-bound to the LTCC at or near the IQ motif (Erickson et al., 2001; Pitt et al., 2001). Upon I_Ca_ activation and consequent Ca^2+^ release from the sarcoplasmic reticulum (SR), local [Ca^2+^]_i_ rises, causing Ca^2+^ to bind to CaM and induce inactivation. The details of the CDI process are not totally resolved, and may involve multiple regions of the channel, including the I-II loop that is thought to be key for VDI (Kim et al., 2004; Cens et al., 2006). An intriguing new hypothesis has emerged from detailed studies from the Yue lab (Ben Johny et al., 2013). During diastole, the C-lobe of apoCaM (CaM without any Ca^2+^ bound) would be associated with the IQ domain, and its N-lobe associated with the pre-IQ domain (between the IQ locus and the upstream EF-hand domain). Ca^2+^ binding to the N-lobe of CaM (the faster, low-affinity site) would cause the N-lobe to shift and bind to part of the LTCC N-terminal domain (which they call the NSCaTE module), and thereby trigger N-lobe CDI. Then when Ca^2+^ also binds to the C-lobe of CaM (the higher affinity, slower binding lobe) the C-lobe shifts its binding from the IQ domain to a position just upstream of the Pre-IQ region where the N-lobe had been bound. If Ca^2+^ binds only to the C-lobe (e.g., if the N-lobe is unavailable) then the C-lobe does a similar sort of shift on its own, and mediates C-lobe CDI. For cardiac Cav1.2 channels, overall CDI and C-lobe-CDI are relatively similar, while N-lobe CDI alone was not apparent (Peterson et al., 1999). That differs from some neuronal P/Q, N or R type Ca^2+^ channels, where N-lobe CDI seems to be dominant (Liang et al., 2003).

**Figure 1:**
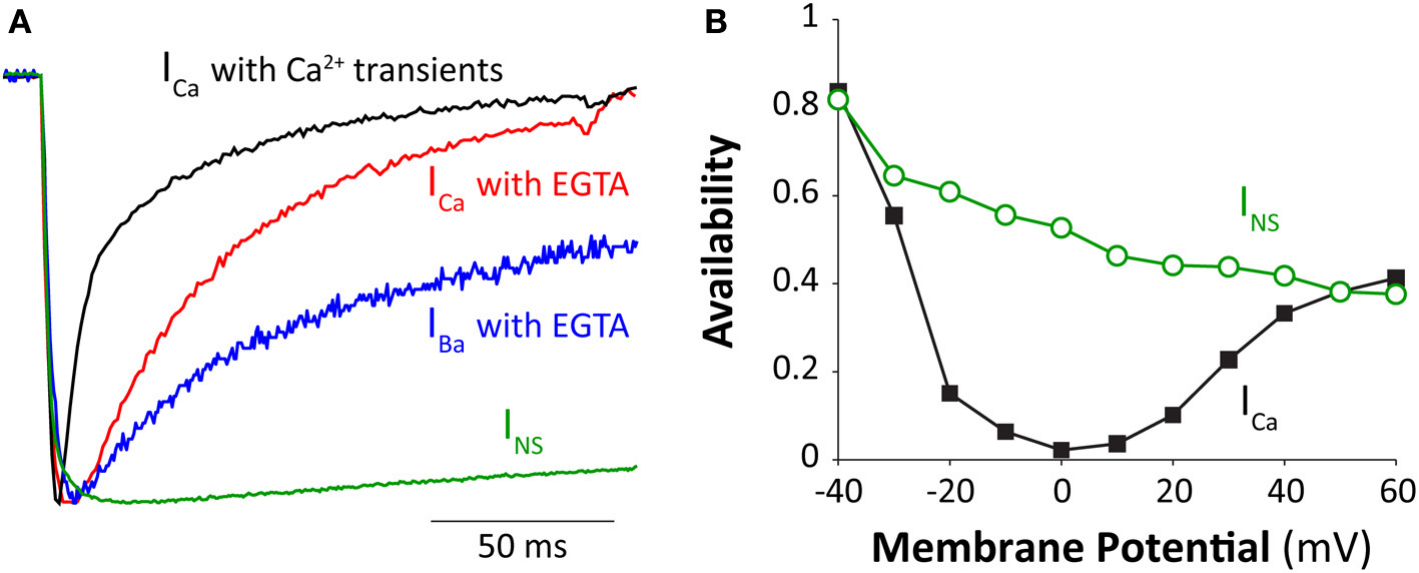
**Inactivation of cardiac Ca^2+^ channel. (A)** Normalized I_Ca_, I_Ba_, and I_NS_ elicited by a square voltage pulse at room temperature to 0 mV (except I_NS_ at −30 mV to obtain comparable activation state). I_Ca_ was recorded under both perforated patch (where normal SR Ca^2+^ release and Ca^2+^ transients occur) and ruptured patch conditions with cells dialyzed with 10 mM EGTA (to prevent global Ca^2+^ transients). I_Ba_ was also recorded with ruptured patch (with 10 mM EGTA in the pipette). Extracellular [Ca^2+^] and [Ba^2+^] were both 2 mM and I_NS_ was measured in divalent-free conditions (10 mM EDTA inside and out) with extracellular [Na^+^] at 20 mM and intracellular [Na^+^] at 10 mM. Peak currents were 1370, 808, 780, and 5200 pA and were attained at 5, 7, 10, and 14 ms for I_Ca_ (perforated), I_Ca_ (ruptured), I_Ba_ and I_NS_ respectively, with t_1/2_ of current decline of 17, 37, 161, and > 500 ms respectively. **(B)** Amplitude of I_NS_ and I_Ca_ through LTCC (at −10 mV) after 500 ms pulses to the indicated E_m_ in guinea-pig ventricular myocytes (modified from Bers, 2001 with permission, data from Hadley and Hume, 1987).

Figure 1A shows I_Ca_ inactivation kinetics in a rabbit ventricular myocyte under different Ca^2+^ conditions. The time to half inactivation (t_1/2_) increases from 17 to 37 ms when normal Ca^2+^ transients are abolished (e.g., by buffering the intracellular Ca^2+^ with 10 mM EGTA). Note that EGTA is a relatively slow buffer and cannot abolish very local [Ca^2+^] elevation around the mouth of the channel (although in this case SR Ca^2+^ release is prevented). In absence of extracellular Ca^2+^, LTCC are permeable to Ba^2+^, and this current (I_*Ba*_) has been often studied to differentiate VDI and CDI (Lee et al., 1985; Peterson et al., 2000; Cens et al., 2006), despite a modest ability of Ba^2+^ to induce inactivation (Ferreira et al., 1997). When Ba^2+^ is the charge carrier (and intracellular Ca^2+^ is buffered), I_Ba_ inactivation is further slowed (*t*_1/2_ = 161 ms).

In the absence of divalent ionic species, LTCC is permeable to monovalent cations and is referred to as non-specific monovalent current (I_NS_, mostly carried by Na^+^ and Cs^+^). I_NS_ inactivates only very slowly at this voltage at room temperature (*t*_1/2_ > 500 ms; Figure 1A), but exhibits VDI, which becomes faster at more positive voltages (Hadley and Hume, 1987; Grandi et al., 2010). I_NS_ inactivation is incomplete (after 500 ms) even at more positive E_m_ (Figure 1B). The additional I_Ca_ inactivation at intermediate E_m_ has an U-shaped E_m_-dependence (as does inward I_Ca_ amplitude, maximal at about 0 mV), reflecting the contribution of CDI. Note that at +50–60 mV little Ca^2+^ enters during I_Ca_, and the extent of I_Ca_ and I_NS_ inactivation is similar. It is tempting to speculate that I_NS_ inactivation properties might provide pure VDI characteristics that are relevant for I_Ca_. However, I_NS_ can actually inactivate faster than I_Ba_ at positive voltages, so we think that using I_NS_ to assess VDI characteristics for I_Ca_ is likely to be invalid (Grandi et al., 2010). However, I_Ba_ inactivation is also not purely VDI, because inactivation is I_Ba_-amplitude dependent (Brunet et al., 2009) and Ba^2+^ can partially substitute for Ca^2+^ in CDI (Ferreira et al., 1997). To resolve this we have attempted to carefully account for the weak Ba^2+^-dependent inactivation and refine the characteristics of VDI vs. CDI in cardiac myocytes in a computational analysis (Morotti et al., 2012). That is, most prior work using I_Ba_ to characterize VDI had slightly overestimated VDI. This is certainly not meant to discourage the use of I_Ba_ vs. I_Ca_ as a means to study CDI, just that this I_Ba_ is not entirely devoid of divalent-dependent inactivation.

Given the role of I_Ca_ in sustaining the AP plateau, CDI and VDI are important determinant for AP duration (APD) regulation. Inhibition of I_Ca_ inactivation induces AP prolongation, and has pro-arrhythmic consequences (see section “Arrhythmogenic consequences of CaMKII-dependent I_Ca_ effects”). For example, impaired VDI has been observed in Timothy syndrome (Splawski et al., 2004, 2005; Brunet et al., 2009), an inherited disease characterized by severe ventricular arrhythmias and sudden cardiac death. The expression of mutant Ca^2+^-insensitive CaM (via adenovirus) in adult guinea-pig cardiomyocytes also prevents CDI and causes dramatic AP prolongation (Alseikhan et al., 2002). Moreover, some human patients with arrhythmias resembling long QT syndrome have linked mutations in the Ca^2+^ binding domains in one of the three CaM genes (which otherwise encode the identical CaM protein; Crotti et al., 2013). A loss of CDI also characterizes the more common pathologic condition of heart failure (HF), where marked AP prolongation and associated defective Ca^2+^ cycling have been reported (Beuckelmann et al., 1992). It is interesting to note that, at first, the down-regulation of repolarizing K^+^ currents (I_to_ and I_K1_) was thought to be responsible for the increased APD seen in HF. Only in the late 1990s the pivotal role of CDI became clear, when it was first proposed in a theoretical study in dog (Winslow et al., 1999), and then experimentally observed in a guinea pig model of HF (Ahmmed et al., 2000). So clearly defective I_Ca_ CDI can be arrhythmogenic in people.

## I_Ca_ during the AP changes with increasing frequency and Ca^2+^ loading

APD regulation is fundamental to control the Ca^2+^ level in myocytes, which is functionally important with respect to the Ca^2+^ requirements for myofilament activation, and thus contractility. Indeed, CDI is a physiological negative feedback mechanism that limits excessive Ca^2+^ entry in myocytes. When the myocyte has relatively high Ca^2+^ load, a large Ca^2+^ transient enhances I_Ca_ inactivation (limiting further Ca^2+^ influx). Conversely, when myocyte Ca^2+^ is low and SR Ca^2+^release is small, there is less CDI and enhanced Ca^2+^ entry that increases intracellular Ca^2+^ content (Puglisi et al., 1999; Eisner et al., 2000; Bers and Grandi, 2009). Notably, Na^+^/Ca^2+^ exchange also participates in this negative feedback (i.e., higher Ca^2+^ transients limit Ca^2+^ entry and increase Ca^2+^ extrusion from the myocyte via Na^+^/Ca^2+^ exchange).

**Figure 2:**
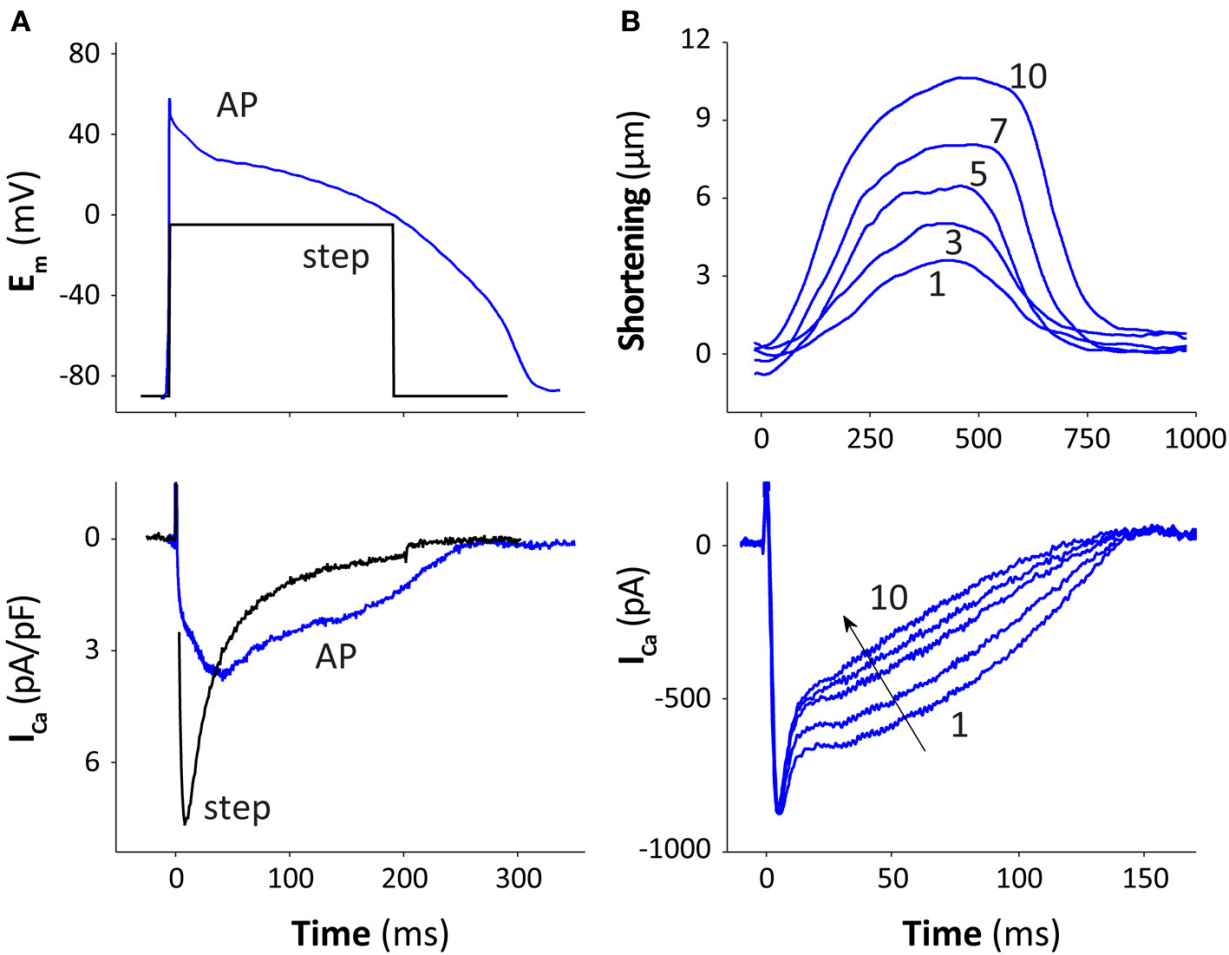
**I_Ca_ inactivation during the AP. (A)** Rabbit ventricular myocytes (at 25°C) were voltage-clamped with either a square voltage step or an AP waveform (measured from 5 other cells under physiological conditions). All other currents were blocked, e.g., by replacement of K^+^ with Cs^+^ and Na^+^ with TEA (inside and out) and cells were dialyzed with 10 mM EGTA to prevent Ca^2+^ transients (data from Yuan et al., 1996, modified from Bers, 2001 with permission). **(B)** After SR Ca^2+^ was depleted by a brief caffeine-application (with Na^+^), a series of AP-clamps were given, and contraction and I_Ca_ recovered to steady state over 10 sequential pulses at 25°C in rabbit ventricular myocyte (modified from Bers, 2001 with permission, data from Puglisi et al., 1999).

The time course of I_Ca_ during the AP is significantly different compared to that seen during a square voltage pulse [Figure 2A, rabbit ventricular myocyte, 25°C, with 10 mM EGTA to prevent Ca^2+^ transients (Yuan et al., 1996)]. Peak I_Ca_ during the AP is lower and occurs later than during a square pulse, with larger I_Ca_ late in the AP. The later I_Ca_ peak is because at the AP peak (+50 mV) Ca^2+^ channels activate rapidly, but the driving force for Ca^2+^ (E_m_–E_Ca_) is initially low, because E_m_ is close to the reversal potential for I_Ca_ (E_Ca_ ~ +60 mV). As E_m_ repolarizes, the driving force increases faster than channel inactivation, producing a larger current at later times during the AP (Sah et al., 2002). Sipido et al. (1995) first investigated how Ca^2+^ released from the SR modulates I_Ca_ performing “classic” voltage-clamp experiments, and observed that CDI increases as SR Ca^2+^ release gets larger. Our group confirmed this observation in a more “physiological” condition, as shown in Figure 2B, where repeated AP-clamps are performed as the SR Ca^2+^ stores are reloaded, such that contractions get progressively larger (beat 1–10; Puglisi et al., 1999). One can see the contribution of SR Ca^2+^ release to CDI as the Ca^2+^ transients and contractions get larger. Integration of the Ca^2+^ influx via I_Ca_ during these ten pulses (which approach the steady state) shows that the I_Ca_-dependent influx decreases from 12 to 6 μmol/L cytosol, indicating that I_Ca_ inactivation due to SR Ca^2+^ release decreases net Ca^2+^ influx by about 50%. These experiments were done at both 25 and 35°C. At 35°C peak I_Ca_ occurs earlier and is higher, but also inactivates faster and the AP duration is also shorter. The net result is that there is very little difference between these temperatures for the integral of Ca^2+^ influx during the AP (with SR Ca^2+^ release fully functional).

Using a combination of AP and square voltage-clamp protocols, Linz and Meyer (1998) assessed the time-course of I_Ca_ inactivation during the AP in different Ca^2+^ homeostasis conditions. Their analysis pointed out that, in physiological condition, CDI is the overwhelmingly dominant inactivation on the time scale of an AP, as recapitulated in the theoretical study by Greenstein and Winslow (2002). Moreover, Linz and Meyer (1998) showed that CDI is mostly controlled by Ca^2+^ released from the SR during the initial part of the AP, then by Ca^2+^ entered through the LTCCs. These results are well described by our recent computational study that updated the balance of VDI and CDI in the context of a detailed Ca^2+^ cycling electrophysiological myocyte model (Morotti et al., 2012).

At increased heart rates, there is typically an increase in Ca^2+^ transient amplitude (known sometimes as the positive force-frequency relationship) in normal hearts in species other than rat and mouse (Bers, 2001). The higher Ca^2+^ transients also typically decline faster at high heart rates (known a frequency-dependent acceleration of relaxation; Bers, 2001). Thus, I_Ca_ inactivation is expected to be faster, based on the above discussion. The higher heart rate could also shorten the diastolic interval and increase diastolic [Ca^2+^]_i_, which might reduce I_Ca_ availability. Indeed, while I_Ca_ recovery from inactivation is classically time and E_m_-dependent (Hadley and Hume, 1987), we showed that elevations of [Ca^2+^]_i_ could slow recovery from inactivation, especially under conditions where SR Ca^2+^ uptake is depressed and diastolic E_m_ is slightly depolarized (Altamirano and Bers, 2007), as can be the case in human HF (Sipido et al., 1998). This sort of diastolic [Ca^2+^]_i_ effect on LTCC availability is probably of only minor relevance under normal physiological conditions and heart rates in healthy hearts, but may be more of a factor under pathophysiological conditions. That is, in HF there is an increased likelihood that peak I_Ca_ will decrease at high heart rates, and that might contribute to limiting the more negative force-frequency relationship observed in HF (Sipido et al., 1998).

## I_Ca_ facilitation is CaMKII-dependent

### Ca^2+^-dependent facilitation of I_Ca_: early functional characteristics

**Figure 3:**
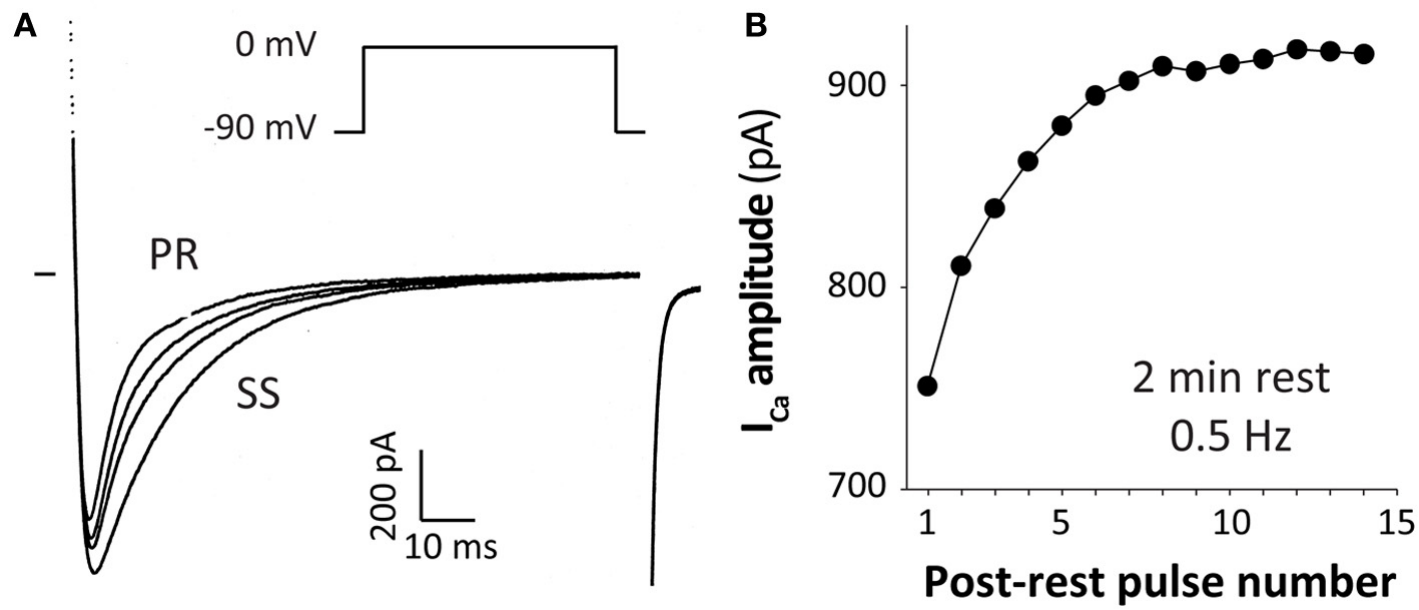
**Ca^2+^-dependent facilitation of I_Ca_.** I_Ca_ at 0.5 Hz (from −90 to 0 mV) after a 2 min rest period in a ferret ventricular myocyte. The first post-rest pulse (PR) and the second, third and steady state (SS) pulses are shown in **(A)** and the whole post-rest I_Ca_ “staircase” in **(B)** (modified from Bers, 2001 with permission, data from Hryshko and Bers, 1990).

Several early studies reported progressive increases in I_Ca_ amplitude and prominent slowing of inactivation that was observed during increased frequency of voltage-clamp pulses from physiological holding potentials (~ −80 mV), as shown in the example in Figure 3 (Lee, 1987; Boyett and Fedida, 1988; Tseng, 1988; Hryshko and Bers, 1990). This phenomenon is not reproduced if holding E_m_ is more depolarized (e.g., −40 mV) where a negative staircase is observed, or in the absence of Ca^2+^ (e.g., when Ba^2+^ is the charge carrier). This I_Ca_ staircase was also stronger when local Ca^2+^ influx was amplified by SR Ca^2+^ release. Thus, this phenomenon is termed Ca^2+^-dependent facilitation of I_Ca_.

CDF and CDI co-exist under physiological conditions, and this may be why I_Ca_ facilitation was masked by holding E_m_ near −40 mV. That is, recovery from inactivation at that E_m_ is slow, so the records were dominated by a negative I_Ca_ staircase that was attributable to CDI and incomplete I_Ca_ recovery from inactivation. It has been proposed that the facilitatory mechanism may partly offset reduced Ca^2+^ channel availability at high heart rates (caused by direct CDI), contributing to improving cardiac performance during exercise (Ross et al., 1995). While CDI responds rapidly (in response to local [Ca^2+^]_i_ during the same beat), CDF occurs more slowly (over several beats). Indeed, biphasic effects of [Ca^2+^]_i_ on unitary I_Ca_ have been reported (Hirano and Hiraoka, 1994). Some studies even claimed that progressive decrease in SR Ca^2+^ release (negative staircase in rat) and CDI are responsible for the observed CDF (Guo and Duff, 2003, 2006). However, because CDF is quite similar in species that exhibit positive Ca^2+^ transients staircases and even when SR Ca^2+^ release is blocked this seems unlikely to be the case (Hryshko and Bers, 1990).

### CDF is CaMKII-dependent: mechanistic studies

**Figure 4:**
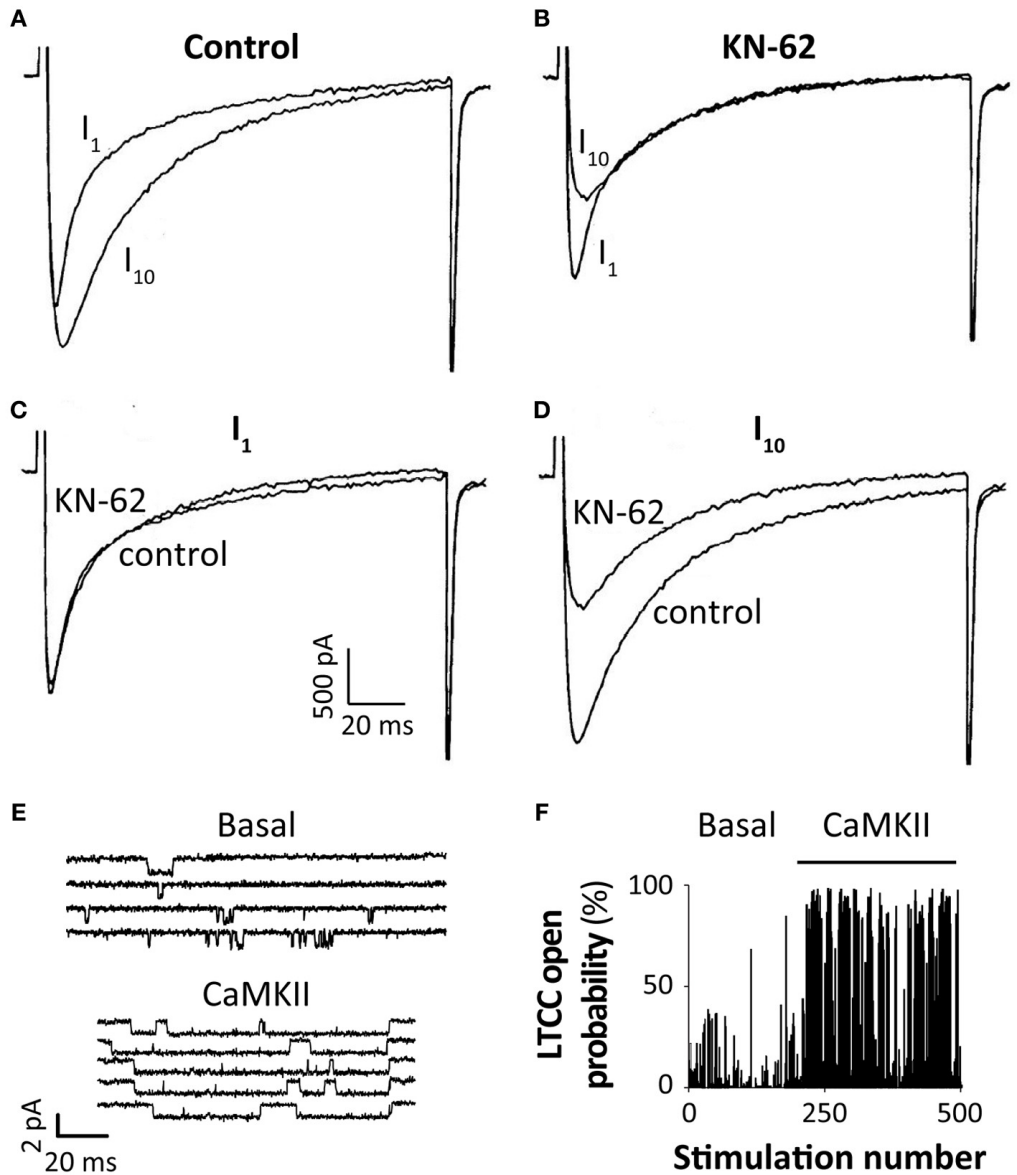
**CaMKII-dependent regulation of I_Ca_.** Superimposed I_Ca_ traces from the first (I_1_) and tenth (I_10_) voltage-clamp pulse from −90 to 0 mV at 2 Hz in a single rabbit ventricular myocyte obtained in control condition **(A)** or after 10 min equilibration with the CaMKII inhibitor KN-62 (1 μM) **(B)**; I_1_ and I_10_ obtained in the two conditions are respectively shown (superimposed) in panels **(C,D)** (modified from Yuan and Bers, 1994 with permission). **(E)** A single LTCC current (channel openings are seen as downward deflections from baseline) is elicited by repetitive depolarizing voltage-clamp steps (from −70 to 0 mV) and reveals infrequent, brief openings under basal conditions (upper panel). CaMKII (bottom) causes frequent and prolonged LTCC openings compared with baseline. Panel **(F)** shows that the probability of LTCC opening during a depolarizing voltage-clamp step is dramatically increased upon addition of CaMKII, compared with basal conditions (modified from Anderson, 2004 with permission, data from Dzhura et al., 2002).

About 20 years ago three groups independently demonstrated that Ca^2+^-dependent I_Ca_ facilitation is mediated by CaMKII-dependent phosphorylation of LTCC (Anderson et al., 1994; Xiao et al., 1994; Yuan and Bers, 1994). Xiao et al. (1994) also observed that sarcolemmal CaMKII activation correlates qualitatively with the changes in I_Ca_. All three studies reported that pharmacological inhibition of CaMKII abolishes CDF in mammalian cardiomyocytes (Figures 4A–D). Anderson's group extended this work by characterizing the CaMKII-dependent effect on single channel I_Ca_ recorded in excised inside-out patches (Dzhura et al., 2000). They showed that addition of activated CaMKII to the cytoplasmic side of the sarcolemma results in phosphorylation of the LTCC complex, inducing high-activity (mode 2) gating that is characterized by long frequent openings (Figures 4E,F), consistently with the macroscopic effect of CDF.

Since CDF is observed when cells are dialyzed with 10 mM EGTA (but is abrogated by 20 mM BAPTA), the active CaMKII must be highly localized near the channels (Hryshko and Bers, 1990). Although the CaMKII-dependent phosphorylation of LTCC has been studied for a long time, the molecular bases of this phenomenon are not still completely understood. In particular, it is debated which LTCC subunit is involved, since multiple candidate phosphorylation sites have been identified in both the pore-forming α1C subunit and the auxiliary β2 subunit (Sun and Pitt, 2011).

Some early studies suggested that the IQ motif on the α1C subunit is involved in CDF (Wu et al., 2001). Wu et al. showed that in rabbit ventricular myocytes I_Ca_ facilitation could be nearly abolished by the CaMKII inhibitory peptide AC3-I, but could then be rescued by cell dialysis with a peptide resembling the Ca^2+^ channel IQ domain, called “IQ-mimetic peptide.” This may also relate to early studies of CDI with wild-type and mutant α1C in Xenopus oocytes, where it was found that isoleucine point mutations in the IQ domain could either enhance (Ile to Ala) or abolish (Ile to Glu) CDF (Zuhlke et al., 1999).

More recent studies in heterologous cells indicate that CaMKII may directly bind and phosphorylate the α1C subunit. In oocytes CaMKII could phosphorylate the α1C subunit (Hudmon et al., 2005). Hudmon et al. (2005) also showed that tethering of CaMKII to the Cav1.2 C-terminus is an essential molecular feature of CDF, because mutations to a putative C-terminus binding site prevent CDF. Other recent studies support the idea of CaMKII-dependent phosphorylation of the pore-forming α1C subunit, and propose possible phosphorylation sites. Erxleben et al. (2006) studied the increase in mode 2 activity of rabbit Cav1.2 channels seen in neurons in two pathologic conditions of cyclosporin neurotoxicity and Timothy syndrome. They found that mode 2 activity increases through a CaMKII-dependent mechanism involving respectively Ser-1517 (at the end of the S6 helix in domain IV), and Ser-439 (at the end of the S6 helix in domain I). Wang et al. (2009) expressed guinea pig Cav1.2 channel in Chinese hamster ovary, and found that CaMKII phosphorylates Thr-1603 residue (Thr-1604 in rabbit) within the pre-IQ region in the C-terminal tail of the Cav1.2 channel. In HEK cells I_Ca_ facilitation was decreased by the single mutations (to Ala) in Ser-1512 and Ser-1570 (two serines that flank the C-terminal EF-hand motif), and abolished by the double mutation S1512A/S1570A (Lee et al., 2006). Furthermore, Blaich et al. (2010) observed impaired I_Ca_ facilitation in mice with knockin mutations at the Ser-1512 and Ser-1570 (to Ala) phosphorylation sites, and confirmed that Cav1.2 channel is modulated by CaMKII-dependent phosphorylation in the murine heart.

In contrast to that data implicating sites on the pore-forming α1 subunit, other results point to CaMKII-dependent phosphorylation of regulatory β subunits. In particular, it was reported that CDF is mediated by phosphorylation of the β2a subunit, at Thr-498 in isolated adult rat (Grueter et al., 2006) and rabbit (Koval et al., 2010) ventricular myocytes. Grueter et al. (2006) first investigated whether, and in which conditions, CaMKII can directly bind to a β2a subunit (expressed as a glutathione S-transferase, GST, fusion protein). They found such high affinity binding when CaMKII was in the active (i.e., autophosphorylated) state. By screening a library of GST-fusion proteins, they identified the β2a region that bound to CaMKII, and verified that CaMKII would phosphorylate this region. Among the different possible phosphorylation sites present in this region, only the mutation of Thr-498 to Ala (T498A) impaired CaMKII-phosphorylation. Expressing T498A β2a with Cav1.2 in tsA201 cells resulted in impaired CaMKII-dependent increase in channel open probability, and ablation of CaMKII-mediated whole cell I_Ca_ facilitation has been observed in rat cardiomyocytes (Grueter et al., 2006). It was also shown that Leu-493 present in the β2a and β1a (but not present in β3 and β4) subunits was important for high affinity CaMKII binding, and that mutation of Leu-493 to Ala (L493A) substantially reduced CaMKII binding, but did not interfere with β2a phosphorylation at Thr-498 (Grueter et al., 2008; Abiria and Colbran, 2010). Other studies have shown that overexpression of β2a, which can dramatically increase I_Ca_, causes cellular Ca^2+^ overload, and facilitates arrhythmogenesis, apoptosis and hypertrophic signaling (Chen et al., 2005; Koval et al., 2010; Chen et al., 2011). Koval et al. (2010) showed that prevention of intracellular Ca^2+^ release by ryanodine, by inhibition of CaMKII activity, or expression of β2a T498A or L493A mutants could reduce Ca^2+^ entry and improved cell survival.

Despite much effort aimed at the detailed molecular mechanism for CaMKII-dependent I_Ca_ facilitation, more work will be required to develop a fully satisfying explanation. It may be that sites on both the α and β subunit are important, that the α−β subunit interaction is critical, and there may also be more than one CaMKII binding domain and phosphorylation target. The CaM involved in activating the CaMKII that is associated with the LTCC seems unlikely to be the same CaM that is involved in CDI, since that CaM appears dedicated and bound strongly even at low [Ca^2+^]_i_ not to fully dissociate from the CDI regulatory sites.

## Arrhythmogenic consequences of CaMKII-dependent I_Ca_ effects

CaMKII-dependent modulation of I_Ca_ is characterized by both increased current amplitude and slowed inactivation, and can result in an overall increase in Ca^2+^ entry, which can be pro-arrhythmic. Intracellular Ca^2+^ overload is associated with increased propensity of spontaneous SR Ca^2+^ release, which can lead to delayed afterdepolarizations (DADs) because of the transient inward current carried by the Na^+^/Ca^2+^ exchanger (in the Ca^2+^ extrusion mode). In a theoretical study (Morotti et al., 2012), we also showed that, when CDI is dramatically impaired, the same mechanism can be responsible for the development of early afterdepolarizations (EADs) during the prolonged AP plateau. It has also been shown that the CaMKII-dependent shift of LTCC into mode 2 gating can explain the global I_Ca_ facilitation typically measured (Hashambhoy et al., 2009). That group also showed that higher mode 2 activity can favor the development of EADs because of I_Ca_ reactivation during the AP plateau (Tanskanen et al., 2005; Hashambhoy et al., 2010). For a further detailed review about mathematical modeling of CaMKII-mediated regulation of LTCC see the accompanying article in this series by Greenstein et al. (2014).

Studying different conditions in which the AP is forcibly prolonged, Anderson's group obtained the first experimental evidence for the role of CaMKII in the development of afterdepolarizations in rabbit ventricular myocytes. They showed that the development of EADs (due to I_Ca_ reactivation during the prolonged plateau) is prevented by CaMKII inhibition (with KN-93 or AC3-I) (Anderson et al., 1998; Wu et al., 1999a), and that AC3-I also prevents the development of DADs caused by the increased Na^+^/Ca^2+^ exchanger current (Wu et al., 1999b). They observed the development of EADs due to CaMKII-dependent enhancement of LTCC open probability in a transgenic mouse model of cardiac hypertrophy as well (Wu et al., 2002). This model, together with increased CaMKII, showed an increased propensity for ventricular arrhythmias, which can be prevented by CaMKII-inhibition. Increased CaMKII levels have been observed also in a murine model of pressure overload HF (Wang et al., 2008). In this model, CaMKII-dependent activation of I_Ca_ is already maximal and CDF cannot be induced, suggesting an important role of CaMKII in remodeling in failing myocytes.

It is now well known that CaMKII is hyperactive in several forms of cardiac diseases (Anderson et al., 2011; Swaminathan et al., 2012; Vincent et al., 2014), and interesting insights about I_Ca_ modulation have been provided by studies on animal models in which CaMKII is overexpressed or inhibited. Both chronic CaMKII overexpression in transgenic mouse myocytes and acute overexpression in rabbit myocytes cause increase in I_Ca_ amplitude and slowing in inactivation (consistent with CDF), and I_Ca_ could be reduced back to control levels by blocking CaMKII with KN-93 or AIP (Maier et al., 2003; Kohlhaas et al., 2006). Conversely, two different mouse models with CaMKII inhibition (Zhang et al., 2005; Picht et al., 2007) are characterized by complete inhibition of I_Ca_ facilitation. Notably, Picht et al. used a CaMKII inhibitory peptide (AIP) genetically targeted to the SR, consistent with the notion that CaMKII involved in I_Ca_ facilitation being localized at junctions between the SR and sarcolemma. Interestingly, Xu et al. (2010) showed that I_Ca_ facilitation was significantly reduced in a CaMKII-knockout mouse model. They also found an increase in Cav1.2 expression, which may be due to a compensatory mechanism for the reduced CaMKII-dependent facilitation over the long-term CaMKII inhibition.

In fact, other studies suggest that CaMKII activity can influence LTCC expression (Meffert et al., 2003; Shi et al., 2005; Ishiguro et al., 2006), based on the evidence that CaMKII phosphorylates the nuclear factor-kappaB (NFkB) component p65, causing its nuclear translocation, and consequent release of NFkB-dependent inhibition of Cav1.2 channel expression. Xu et al. (2010) found a significant reduction of p65 nuclear translocation in their transgenic myocytes.

Beyond LTCC, CaMKII influences many other targets within the cell (Bers and Grandi, 2009), many of which play important roles in modulating the cardiac ECC. An accurate analysis of the arrhythmogenic consequences of CaMKII-dependent LTCC phosphorylation cannot neglect, among the various targets, the effects on phospholamban (PLB) and ryanodine receptors (RyRs). CaMKII phosphorylation of PLB releases its inhibition on Ca^2+^-sensitivity of SR Ca^2+^ pump (Simmerman and Jones, 1998), thus causing an increase in the pump affinity for Ca^2+^. When RyRs are phosphorylated, their sensitivity for cytosolic Ca^2+^ (Li et al., 1997; Wehrens et al., 2004) and passive leak (Ai et al., 2005; Guo et al., 2006) are enhanced. Thus, consequences of CaMKII-dependent phosphorylation of RyRs and PLB are increased SR Ca^2+^ uptake and release, resulting in an increase in Ca^2+^ transient amplitude, which further activates CaMKII, and this can have arrhythmogenic consequences. Integrated mathematical models have been helpful in quantitatively understanding the complex interactions among these players. Soltis and Saucerman (2010) demonstrated the key role of RyR phosphorylation in the prominent positive feedback that associates the CaMKII-dependent increase in Ca^2+^ signal to a further increase in CaMKII activity. They also showed that the CaMKII-Ca^2+^-CaMKII feedback is enhanced by β-adrenergic stimulation (which further enhances Ca^2+^ signal). We recently extended their work, by studying the synergy of Na^+^ handling with Ca^2+^ and CaMKII signaling, since CaMKII hyperactivity in HF has also been associated with late I_Na_ and intracellular [Na^+^] ([Na^+^]_i_) overload (Wagner et al., 2006; Grandi and Herren, 2014). We found that a significant gain in [Na^+^]_i_ (~ 3–4 mM), which is what happens in HF (Despa et al., 2002), induces an increase in Ca^2+^ and consequent Ca^2+^-dependent CaMKII activation, which in turn enhances Na^+^ and Ca^2+^ signals, leading to a pro-arrhythmic condition. We also showed that, in condition of CaMKII overexpression, the CaMKII-Na^+^-Ca^2+^-CaMKII feedback is predominant, and leads to a hyper-phosphorylation of RyRs responsible for spontaneous SR Ca^2+^ release and DADs development (Morotti et al., 2014).

## Concluding remarks

CaMKII has numerous targets in cardiac myocytes, and we must assume that under normal physiological conditions this orchestrates a response that is acutely adaptive. However, when CaMKII becomes chronically activated in disease, by autophosphorylation and oxidation (Anderson et al., 2011; Swaminathan et al., 2012), O-GlcNAcylation (Erickson et al., 2013) or possibly nitrosylation (Gutierrez et al., 2013), these regulatory systems may become maladaptive. The key CaMKII-dependent regulation of LTCC is I_Ca_ facilitation, a moderate increase in I_Ca_ amplitude and slowing of I_Ca_ inactivation in response to changes in heart rate. It seems likely that I_Ca_ facilitation is a normal adaptation to increased heart rate, to ensure Ca^2+^ channel availability and the integrity of ECC (which might otherwise be depressed by CDI or encroachment into recovery from inactivation). However, when this system is chronically on in pathological states it may contribute to inappropriate Ca^2+^ loading of the myocytes, and contribute to worsening pathology via poor diastolic function or arrhythmias triggered by EADs or DADs, altered I_Ca_ restitution or cardiac alternans. The detailed molecular mechanisms remain to be fully resolved, but work over the past 10–20 years has paved the way for further clarification in the near future.

### Conflict of interest statement

Donald M. Bers received a research grant from Gilead Sciences in May 2013. Gilead Sciences was in no way involved in the design, funding, execution, or interpretation of this study. Stefano Morotti has nothing to disclose.
